# Metformin therapy and risk of colorectal adenomas and colorectal cancer in type 2 diabetes mellitus patients: A systematic review and meta-analysis

**DOI:** 10.18632/oncotarget.13762

**Published:** 2016-12-01

**Authors:** Feifei Liu, Lijing Yan, Zhan Wang, Yuanan Lu, Yuanyuan Chu, Xiangyu Li, Yisi Liu, Dongsheng Rui, Shaofa Nie, Hao Xiang

**Affiliations:** ^1^ Department of Epidemiology and Biostatistics, School of Public Health, Wuhan University, Wuhan, 430071, China; ^2^ Global Health Institute, Wuhan University, Wuhan, 430071, China; ^3^ Global Health Research Center, Duke Kunshan university, Kunshan, Jiangsu, 215316, China; ^4^ Environmental Health Laboratory, Department of Public Health Sciences, University Hawaii at Manoa, Honolulu, HI 96822 USA; ^5^ Department Of Public Health, Medicial College Shihezi University, Shihezi city, 832000, China; ^6^ Department of Epidemiology and Biostatistics and MOE Key Lab of Environment and Health, School of Public Health, Tongji Medical College, Huazhong University of Science and Technology, Wuhan, 430030, China

**Keywords:** metformin, colorectal adenomas, colorectal cancer, type 2 diabetes mellitus, meta-analysis

## Abstract

Recent evidence indicates that metformin therapy may be associated with a decreased colorectal adenoma/colorectal cancer risk in type 2 diabetes patients. However, results are not consistent. We therefore performed a systematic review and meta-analysis to assess the association between metformin therapy and risk of colorectal adenomas/colorectal cancer in type 2 diabetes mellitus patients. We searched the literature published before Aug 31, 2016 in four databases: PubMed, Embase database, CNKI and VIP Library of Chinese Journal. Summary risk estimates (adjusted OR/adjusted RR/adjusted HR) with their 95% confidence interval (95% CI) were obtained using a random effects model. Twenty studies (including 12 cohort studies, 7 case-control studies and 1 randomized controlled trial study) were selected in terms of data of colorectal adenomas or colorectal cancer incidence. Metformin therapy was found to be associated with a decreased incidence of colorectal adenomas (unadjusted OR=0.80, 95% CI: 0.71-0.90, *p*=0.0002). When the adjusted data were analyzed, the summary estimate decreased to 25% reduction in colorectal adenomas risk (adjusted OR=0.75, 95% CI: 0.59-0.97, *p*=0.03). Besides, a significant reduction of colorectal cancer risk was also observed (unadjusted OR=0.73, 95% CI: 0.62-0.86, *p*=0.0002). And when the adjusted data were analyzed, colorectal cancer risk for metformin users was decreased with a reduction of 22%, compared with non-metformin users and other treatment users (adjusted OR=0.78, 95% CI: 0.70–0.87, *p*<0.00001). Our meta-analysis suggested that metformin therapy may be associated with a decreased risk of colorectal adenomas and colorectal cancer in type 2 diabetes mellitus patients.

## INTRODUCTION

According to WHO, over 220 million people are suffering from diabetes disease. The International Diabetes Federation estimates that the number of people with impaired glucose tolerance will increase to 472 million (9 % of the adult population) by 2025, and around 40-50% of them will develop type 2 diabetes mellitus (T2DM) [1]. Many studies have provided strong evidences that population with diabetes disease is at significantly higher risk of developing many forms of cancer, especially for solid tumors. In addition to pancreatic and breast cancer, high incidence of colorectal cancer was also associated with T2DM [2] for metabolic syndromes, especially insulin resistance, hyperinsulinemia and hyperglycemia independently [3]. However, recent studies proposed that the risk of colorectal cancer may be reduced among T2DM patients accepting metformin therapy [4–6].

Metformin is a potent anti-hyperglycemic agent. It can reduce hyperinsulinemia, improve insulin resistance [7], and lower blood glucose concentrations in T2DM patients without causing hyperglycemia [8]. Besides, metformin can also reduce glucose uptake from the intestinal tract, improve insulin sensitivity and utilization by adipose tissue and skeletal muscle. Now metformin has been recommended as the first line oral therapy for newly diagnosed T2DM by many professional diabetes organizations.

Some researchers indicated that metformin may be a potential protective factor of colorectal adenomas and colorectal cancer in T2DM patients. Kim et al [9] and Cho et al [10] found that metformin was independently associated with decreased colorectal adenomas incidence. Besides, Tseng et al [11] found that metformin can reduce colorectal cancer risk (adjusted RR=0.73, 95% CI: 0.58-0.92, *p*=0.0115). Similar results were also reported in other studies [5, 12, 13]. However, some other studies found no relation between metformin therapy and colorectal adenomas or colorectal cancer [5, 9, 14, 15], thus the results were still inconsistent. Inconsistencies in these results may cause by differences in study design, populations, or different statistical methods. Some researchers have performed systematic reviews and meta-analysis to assess the relationship between metformin use and cancer risk, however, results refer to colorectal cancer were not consistent [16, 17], and the review of association between metformin therapy and colorectal adenomas risk has not been published until now. In summary, it is still inconclusive whether use of metformin could protect T2DM patients from colorectal adenomas and colorectal cancer.

To explore associations between metformin therapy and colorectal cancer and adenomas risk, we identified available studies to make a quantitative meta-analysis for the purpose of more cognition of using metformin in T2DM patients. This investigation is important to identify the existing knowledge on the effects of metformin therapy in T2DM patients.

## RESULTS

### Search results

**Figure 1:**
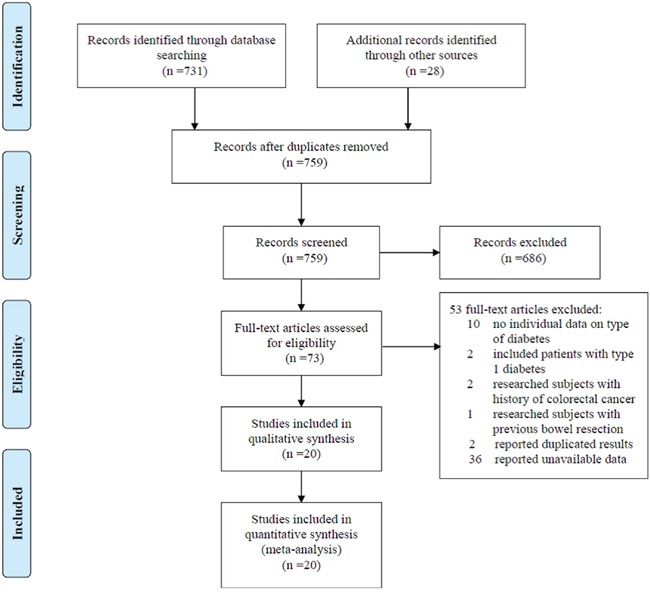
Flow chat of selecting studies for meta-analysis

We identified 759 unique papers after searching PubMed, Embase database, China National Knowledge Infrastructure (CNKI), and VIP Library of Chinese Journal. Based on the inclusion and exclusion criteria defined above, we excluded 739 articles. A total of 20 full-text articles were included in this meta-analysis (Figure 1). Among these articles, twelve were cohort studies [4, 5, 9–13, 18–22], seven were case-control studies [14, 15, 23–27] and one was randomized clinical trial (RCT) study [28]. The main characteristics of the 19 observational studies included in the present analysis are reported in Table 1 and Table 2. All observational studies were population-based. Quality assessment of The Newcastle-Ottawa Scale (NOS) showed that publications that met eligibility were of acceptable quality to be included in these meta-analyses (Supplementary Table 1, Table 2).

**Table 1:** Characteristics for publications included in the meta-analysis of metformin and colorectal adenoma

First author	year	Study type	Region	Period	Treatment		Control		Adjusted OR/RR, 95%CI
					n	N	n	N	
Yong Woo Chung [15]	2008	case–control	Korea	2003-2006	34	100	45	100	0.7(0.3-1.4)
James.D 1[23]	2008	case–control	U.S.	1994-2005	414	1296	1122	2952	-
James.D 2[23]	2008	case–control	U.S.	1994-2005	351	951	3367	8862	-
James.D 3[23]	2008	case–control	U.S.	1994-2005	71	159	833	1666	-
Jain D [27]	2016	case-control	Mixed	-	70	211	36	88	-
Youn Hee Cho [10]	2014	cohort	Korea	2001-2013	139	912	450	2193	0.738(0.554-0.983)
Yo Han Kim [9]	2015	cohort	Korea	2002-2012	80	151	53	89	0.866(0.453-1.623)

**Table 2:** Characteristics for publications included in the meta-analysis of metformin and colorectal cancer

First author	year	Study type	Region	Period	Treatment		Control		Adjusted OR/RR/HR, 95%CI	Adjust Factors/Covariates												
					n	N	n	N		Age	Sex	Smoking	Deprivation	Alcohol use	BMI	HbA1C	Other disease	other drug	CCI score	diabetes duration	metformin duration	T-stage
Gillian Libby[4]	2009	cohort	UK	1994-2003	40	4085	76	4085	0.60(0.38-0.94)	✓	✓	✓	✓		✓	✓		✓				
Meei-Shyuan Lee[18]	2011	cohort	China	2000-2007	30	11221	26	4213	0.36(0.13-0.98)	✓	✓							✓	✓		✓	
Michael Bodmer[24]	2011	case–control	UK	1995-2009	416	920	2354	5519	1.43(1.08-1.90)			✓			✓			✓		✓	✓	
Rikje Ruiter[5]	2012	cohort	Netherlands	1998-2008	228	52698	299	32591	0.91(0.88-0.94)	✓	✓							✓		✓		
Ming-Chia Hsieh 1[12]	2012	cohort	China	2000-2008	46	3963	145	6072	0.54(0.38-0.75)	✓	✓							✓				
Ming-Chia Hsieh 2[12]	2012	cohort	China	2000-2008	46	3963	18	751	0.46(0.27-0.81)	✓	✓											
Chin-Hsiao Tseng[11]	2012	cohort	China	2003-2005	206	26982	472	61009	0.73(0.58-0.92)	✓	✓	✓					✓	✓		✓	✓	
Smiechowski B[25]	2013	case–control	UK	1998-2009	444	607	4406	5837	0.94(0.74-1.19)			✓			✓	✓	✓	✓		✓		
Majken Cardel[14]	2014	case–control	Danish	2000-2009	164	1255	842	5454	0.83(0.68-1.00)	✓	✓	✓		✓	✓			✓			✓	
Amikar Sehdev[26]	2014	case–control	U.S.	2005-2010	983	2682	2059	5364	0.85(0.76-0.95)						✓		✓	✓	✓		✓	
Konstantinos K[19]	2014	cohort	UK	1987-2010	353	51484	246	18264	0.92(0.76-1.13)			✓		✓	✓			✓		✓		
Susan Spillane[20]	2014	cohort	UK	2001-2006	135	241	78	129	0.66(0.39-1.12)													✓
Hua Xu 1[21]	2015	cohort	U.S.	1995-2010	177	2218	63	903	0.50(0.31-0.81)	✓	✓	✓			✓		✓	✓				
Hua Xu 2[21]	2015	cohort	U.S.	1995-2010	212	3029	114	1629	0.60(0.44-0.83)	✓	✓	✓			✓		✓	✓				
Yu-Ching Chen[13]	2015	cohort	China	1998-2007	18	2223	69	3965	0.50(0.30-0.81)	✓	✓	✓	✓	✓	✓		✓					
Bernd Kowall 1[22]	2015	cohort	Germany and UK	1995-2013	281	55988	150	17704	1.05(0.85-1.30)	✓	✓				✓		✓	✓		✓		
Bernd Kowall 2[22]	2015	cohort	Germany and UK	1995-2013	281	55988	26	6571	0.97(0.64-1.46)	✓	✓				✓		✓	✓		✓		

### Metformin therapy and risk of colorectal adenomas

Five studies, including case-control studies [15, 23, 27] and cohort studies [9, 10], provided sufficient data to be included in the meta-analysis to evaluate the association between metformin therapy and the risk of colorectal adenomas among T2DM patients. Table 1 shows characteristics of five studies meeting the inclusion criteria and selected for the quantitative analysis. These studies were published after 2007 and mainly conducted in the United States and Korea. While the other study [23] included three different site adenomas and was analyzed separately.

**Figure 2:**
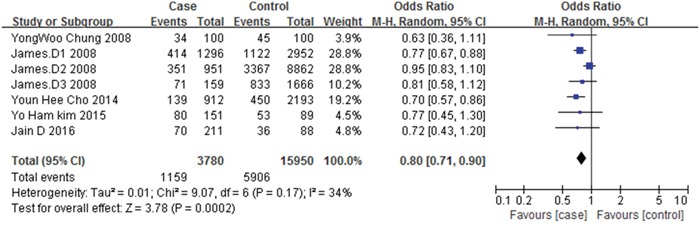
Forest plot of the association between metformin therapy and colorectal adenomas

A pooled estimate of OR and 95% CI of association between metformin therapy and colorectal adenomas among the five studies is shown in Figure 2. Metformin therapy was found to be associated with a decreased incidence of colorectal adenomas (OR=0.80, 95% CI: 0.71-0.90, *p*=0.0002). Low between-study heterogeneity was found (I^2^=34%, *p*=0.17) (Figure 2).

Subgroup analysis was conducted based on the type of study design. A significant reduction of colorectal adenomas risk was observed in both nested case-control studies (unadjusted OR=0.82, 95% CI: 0.72-0.95, *p*=0.006) and retrospective cohort studies (unadjusted OR=0.71, 95% CI: 0.58-0.86, *p*=0.0004) (Supplementary Figure 1).

When only the adjusted OR estimates from 3 studies [9, 10, 15] were analyzed, the summary estimate decreased to 25% reduction in colorectal adenoma risk (adjusted OR=0.75, 95% CI: 0.59-0.97, *p*=0.03), low between-study heterogeneity was found (I^2^=0%, *p*=0.89) (Supplementary Figure 2).

For we only have five studies refer to metformin therapy and colorectal adenomas risk, the number is too small to conduct the Begg's and Egger's test. However, the funnel plot illustrated a symmetrical distribution of the points, suggesting a lack of publication bias (Supplementary Figure 3).

### Metformin therapy and risk of colorectal cancer

Fifteen studies (Four nested case-control studies [14, 24–26], Ten cohort studies [4, 5, 11–13, 18–22] and one RCT [28]) reported the association between metformin therapy and colorectal cancer risk. Table 2 shows characteristics of all observational studies meeting the inclusion criteria and selected for the quantitative analysis. While two studies [12, 22] included two different compare drugs and one studies [21] included different places data of colorectal cancer incidence, data of these three studies were analyzed separately.

**Figure 3:**
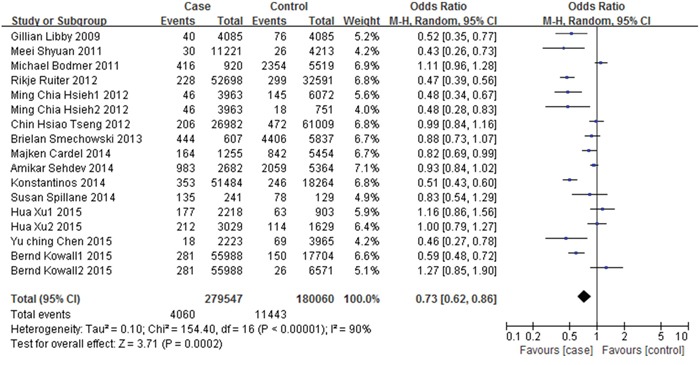
Forest plot of the association between metformin therapy and colorectal cancer - unadjusted odds ratios

Based on data of crude numbers of case and controls, the summary unadjusted odds ratio with the random effects model was 0.73 (95% CI: 0.62-0.86, *p*=0.002) for metformin users (metformin exposure more than 1 year) compared with non-metformin users or other treatment users. High between-study heterogeneity was found (I^2^=90%, *p*<0.00001) (Figure 3).

**Figure 4:**
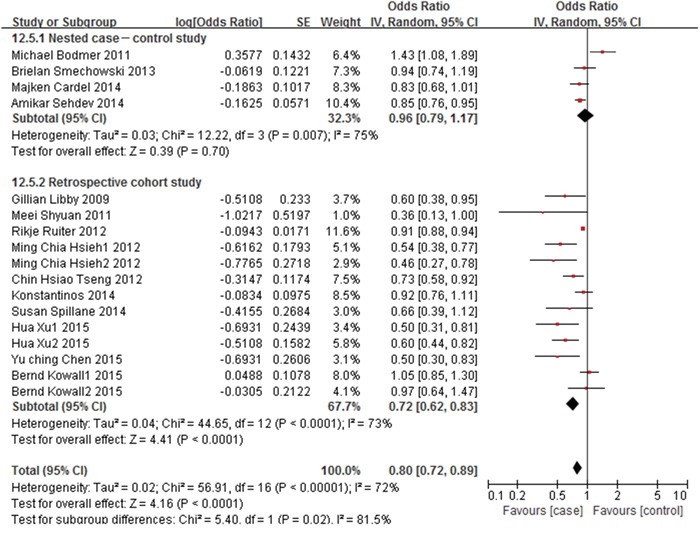
Forest plot of the association between metformin therapy and colorectal cancer - adjusted odds ratios

When the adjusted data estimates from all observational studies were analyzed, the colorectal cancer risk for metformin users was decreased with a reduction of 22%, compared with non-metformin users or other treatment users (adjusted OR=0.78, 95% CI: 0.70–0.87, *p*<0.00001), the between-study heterogeneity was decreased (I^2^=71%, *p*<0.00001) (Figure 4).

Subgroup analysis was conducted based on the type of study design, no significant relationship was found in nested case-control studies (adjusted OR=0.96, 95% CI: 0.79-1.17, *p*=0.70). However, a significant reduction of colorectal cancer risk was observed in retrospective cohort studies (adjusted OR=0.72, 95% CI: 0.62-0.83, *p*<0.0001) (Figure 4).

As to the meta-analysis on the type of risk estimates (adjusted OR/adjusted RR/adjusted HR), a significant reduction of colorectal cancer risk was observed in adjusted OR data (adjusted OR=0.85, 95% CI: 0.78-0.92, *p*=0.0001). High between-study heterogeneity was found (I^2^=80%, *p*=0.001) (Supplementary Figure 4). After omission one of the study [24], the between-study heterogeneity was decreased to 0%. However, no relationship was found with the adjusted RR data (adjusted RR=0.83, 95% CI: 0.65-1.06, *p*=0.13). Moderate between-study heterogeneity was found (I^2^=55%, *p*=0.14) (Supplementary Figure 5). And a significant reduction of colorectal cancer risk was observed in adjusted HR data (adjusted HR=0.77, 95% CI: 0.65-0.91, *p*=0.002). Moderate between-study heterogeneity was found (I^2^=69%, *p*=0.001) (Supplementary Figure 6)

**Figure 5:**
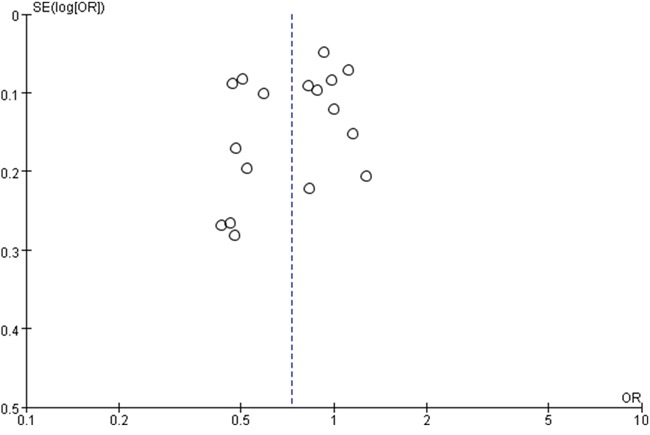
Funnel plot of metformin therapy and colorectal cancer included in meta-analysis

We evaluated the possibility of publication bias in the articles. No obvious publication bias was found by Begg's test (Z=1.52, *p*=0.127) (Supplementary Figure 7) and Egger's test (t= -1.40, *p*=0.182) (Supplementary Figure 8). The funnel plot also illustrated a symmetrical distribution of the points, suggesting a lack of publication bias (Figure 5).

## DISCUSSION

This meta-analysis suggests that metformin therapy may be associated with a decreased risk of colorectal adenomas (unadjusted OR=0.80, 95% CI: 0.71-0.90, *p*=0.0002) and colorectal cancer (adjusted OR=0.70, 95% CI: 0.72-0.89, *p*<0.0001) among T2DM patients, compared with non-metformin users and other diabetic drug users. The degree of heterogeneity among studies may cause by the difference in study design, populations or comparators. However, almost all of the results showed a protective effect of metformin.

Our results are consistent with previous observations suggesting a protective role of metformin for colorectal cancer [16, 17, 29]. In 2011, Zhang et al [17] found the metformin therapy was associated with a significantly lower risk of colorectal cancer (OR=0.63, 95% CI: 0.47-0.84, *p*=0.002). Similarly, Monica Franciosi et al [29] reported that the risk of colorectal cancer was decreased by 17% (OR=0.83, 95% CI: 0.74–0.92, *p*=0.0009). However, Andrea DeCensi et al [16] found an inverse association between metformin use and colon cancer incidence, although not significant (SRR=0.64, 95% CI: 0.38-1.08). Similarly the RCT study of P.D. Home [28], which was included in our search results, also showed no association between metformin treatment and colorectal cancer risk. These conflicting results may cause by methodological problems within studies: the small number of participants in studies may limit the statistical power to obtain significant results. For example, only 50 participants in the metformin group and 55 participants in the comparison group were included in the RCT study [28].

Metformin also showed a protective effect in many other cancers. A meta-analysis conducted by Wu et al [30] showed that metformin therapy was associated with a significant reduced risk of prostate cancer among cohort studies (RR=0.92, 95% CI: 0.87-0.96, *p*<0.001). Zhu et al [32] confirmed that compared with other antidiabetic drugs, metformin was significantly associated with a 16% reduction of lung cancer risk in T2DM patients (RR=0.84, 95% CI: 0.73-0.97, *p*=0.019). And a study by Tseng et al [33] proved metformin use is associated with a decreased risk of kidney cancer in patients with T2DM patients (HR=0.279, 95% CI: 0.254-0.307, *p*<0.0001). These studies confirmed that metformin therapy may serve as a protective factor in decreasing cancer risk.

Evidences from previous studies indicate that metformin may interfere with carcinogenesis through direct (insulin-independent) and indirect (insulin-dependent) mechanisms. Metabolic syndromes in T2DM patients such as insulin resistance, hyperinsulinemia and hyperglycemia are risk factors of several cancers [2, 35, 36]. Metformin can reduce blood glucose directly and can lower insulin resistance and hyperinsulinemia, then may reduce cancer risk among T2DM patients. Meanwhile, cell experiments show that metformin can reduce cell survival by increasing reactive oxygen species, which induce DNA damage and apoptosis [37]. Studies also found metformin can active the intracellular adenosine monophosphate-activated protein kinase (AMPK), which leads to down regulation its downstream target p70S6K (pS6K), resulting in suppression of cellular proliferation, migration and invasion [6, 38]. However, these investigations are still limited by small sample size, and need to be verified repeatedly by experiment.

Compared with other existing studies, our meta-analysis has several advantages. Firstly, our analysis included a much larger number of studies, and provides more precise results. Sensitivity analysis also indicated that our pooled risk estimates were considerably credible. Secondly, we adopted more rigorous criteria for study selection to ensure the reliability of the meta-analysis, taking into account factors which may affect the results. In addition, we analyzed the results separately by type of diseases (colorectal adenoma and colorectal cancer), which provided a stronger evidence of protective effect of metformin among T2DM patients.

However, our study also has several limitations. Firstly, the selection of populations is inconsistent in different studies, which may be an important source of heterogeneity. Secondly, the description of drug dose and length is unclear among studies. Most studies usually classify metformin as two groups: metformin users vs. non-users or other treatment user. And few studies collected metformin dose data. There might have a dose–response relationship between metformin use and cancer incidence for T2DM patients, and we can't calculate it, which may be another large source of high heterogeneity in our studies. Thirdly, different adjustments may influence the summary estimation of the association between metformin treatment and colorectal adenomas/colorectal cancer risk. Furthermore, the protective effect of metformin may be overestimated due to the association between insulin using and higher risk of colorectal cancer [39]. Future studies need to take all these factors into account and provide more precise results estimate for metformin therapy on colorectal cancer incidence.

In conclusion, our meta-analysis indicates that metformin is associated with a reduction in risk of colorectal adenoma and colorectal cancer incidence in individuals with T2DM, compared with other diabetic treatments. Our results also support the hypothesis that in T2DM patients with high risk for colorectal adenomas/colorectal cancer (due to their metabolic condition), the candidates for drug therapy may better be treated with metformin. And further prospective studies, especially large-scale clinical studies and cell experiments are needed to confirm the relationship between colorectal cancers and metformin treatment.

## MATERIALS AND METHODS

Our meta-analysis was in accordance with the Systematic Reviews and Meta-Analysis guidelines: PRISMA Checklist.

### Search strategies

A comprehensive literature search was performed in the PubMed, Embase database, China National Knowledge Infrastructure (CNKI) and VIP Library of Chinese Journal from their earliest available data to Aug 31, 2016. The following search terms and combinations were used in keyword and subject heading search: (“diabetes” or “diabetes mellitus” or “DM” or “insulin resistance“) and (“therapy” or “treatment” or “therapeutics” or “therapeutics” or “metformin” or “biguanides“) and ((“colorectal” or “colon” or “rectal” or “rectum“) or (“neoplasm” or “neoplasia” or “cancer” or “tumor” or “carcinoma” or “adenoma“)). Only human studies were included. The languages of included articles were limited to English and Chinese. A manual search was also conducted for references cited in the selected articles. (refer supplementary for detailed search strategy).

### Inclusion and exclusion criteria

We applied five inclusion criteria on retrieved articles. The eligible paper should: (1) be an original article; (2) report randomized or observational studies; (3) study patients with T2DM ; (4) have metformin therapy as the first treatment (metformin exposure more than 1 year) compared with non-metformin user (never use metformin) and other treatments (insulin, sulfonylurea, thiazolidinedione etc.); (5) provide relative risk (RR), odds ratio (OR) or hazard ratio (HR) with their 95% confidence interval (95% CI), or provide sufficient data to allow adequate estimation of the RR/OR and 95% CI. We dropped the paper if it: (1) cases report, summary, review or meta-analysis; (2) had no individual data on type of diabetes; (3) included patients with type 1 diabetes; (4) researched subjects with complication (diabetic retinopathy etc.), hereditary colorectal syndromes, history of colorectal cancer, chronic inflammatory bowel disease, or previous bowel resection; (5) reported duplicated results or unavailable data.

### Data extraction

Two authors (FFL and HX) selected studies independently according to the inclusion and exclusion criteria listed above. The characteristics extracted were the name of first author, publication year, study design, race, study period, adjust factor or covariance, crude numbers of exposed and unexposed subjects, crude numbers of colorectal adenomas/colorectal cancer in exposed group and unexposed group (for cohort studies), or crude numbers of cases and controls, crude numbers of colorectal adenomas/colorectal cancer in case and control group (for case-control studies), RR, OR or HR with their 95% CI. When there were multiple publications from the same population, only the study with largest sample size were included. Conflicts of data extraction between the two reviewers were resolved by discussion and consensus with an arbitrator (SFN).

### Quality assessment

Two authors (DSR and YSL) conducted quality assessment of included observational studies (cohort study and case-control study) independently according to The Newcastle-Ottawa Scale (NOS)[41]. This scale includes eight items, which are divided into three parts: Selection, Comparability and Outcome. After evaluating these three domains of each individual study, it could be scored a maximum of nine stars. A study earning seven or more stars was seen to be of high quality. Conflicts of the results of quality assessment were resolved by discussion and consensus with an arbitrator (SFN).

### Statistical analysis

In this meta-analysis, we used a random-effects model to estimate the pooled effect across studies for the association between metformin therapy and the risk of colorectal adenomas/colorectal cancer. For the random-effects model accounts for variations between studies in addition to sampling error within studies [42]. We calculated the summary OR, RR and HR with 95% CI with specific adjusted data taken directly from the study or estimated by crude numbers for the association between metformin therapy and colorectal adenomas/colorectal cancer. Heterogeneity among studies was evaluated using I^2^ statistics [43]. I^2^ >75.0%, 50.0–75.0% and I^2^ <50% indicate low, moderate, and high heterogeneity respectively. Sensitivity analysis was conducted to identify studies contributing disproportionately to the observed heterogeneity, by omitting a study at a time to analyze the influence of individual studies on the summary estimate [42]. The significance of the pooled OR, RR or HR was determined by the Z-test, and *p* value less than 0.05 was considered statistically significant. RevMan (Version 5.2) was used for these calculations.

The possibility of publication bias, which may be caused by the non-publication of small number of studies with negative findings, was assessed with a funnel plot for asymmetry. Besides, the Begg's test and Egger's test were also conducted to assess the publication bias [44]. The meta-analysis was considered to have significant publication bias if the *p* value was less than 0.05. These analyses were carried out with Stata (Version 12.0).

## SUPPLEMENTARY MATERIALS FIGURES AND TABLES


