# Supplementary material for: Metformin therapy and risk of colorectal adenomas and colorectal cancer in type 2 diabetes mellitus patients: A systematic review and meta-analysis
Source: Oncotarget. 2016 Dec 1;8(9):16017–26. doi: 10.18632/oncotarget.13762 (PMC5362542; doi:10.18632/oncotarget.13762)
Supplement: Supplementary file 1 [file oncotarget-08-16017-s001.pdf]

Metformin therapy and risk of colorectal adenomas and colorectal cancer in type 2 diabetes mellitus patients: a systematic review and meta-analysis

SUPPLEMENTARY MATERIAL

Search strategy for review of research on metformin treatment and risk of colorectal adenoma and colorectal cancer

Searches on the four databases:

English Databases: Pubmed, Embase

Chinese Databases: CNKI, VIP

PubMed

Search strategy

1. "diabetes mellitus"[MeSH Terms] OR "diabetes"[All Fields]
2. "mellitus"[All Fields] OR "diabetes mellitus"[All Fields] OR "diabetes"[All Fields]
3. 1 and 2
4. "therapy"[Subheading] OR "therapy"[All Fields] OR "treatment"[All Fields]
5. "therapeutics"[MeSH Terms] OR "therapeutics"[All Fields]
6. "metformin"[MeSH Terms] OR "metformin"[All Fields]
7. 4 or 5 or 6
8. "colorectal neoplasms"[MeSH Terms] OR "colorectal"[All Fields] AND "neoplasms"[All Fields] OR "colorectal neoplasms"[All Fields]
9. "colorectal"[All Fields] AND "cancer"[All Fields]) OR "colorectal cancer"[All Fields]
10. colorectal[All Fields] AND poly[All Fields] OR colorectal[All Fields]
11. 8 or 9 or 10
12. "adenoma"[MeSH Terms] OR "adenoma"[All Fields]
13. 11 and 12
14. 3 and 7 and 13

EMBASE

Search strategy

1. 'diabetes mellitus'/exp
2. 'diabetes'/exp OR 'diabetic'/exp OR glycemic OR glycaemic OR 'sugar'/exp OR 'glucose intolerance'/exp OR hyperglycemia:ab,ti
3. #1 OR #2
4. 'metformin'/exp
5. 'biguanide derivative'/exp
6. metformin* OR glucophag* OR biguanid*:ab,ti

12. #4 OR #5 OR #6
13. 'colorectal tumor'/exp
14. rectal:ti OR rectum:ti OR colon*:ti OR colorect*:ti
15. cancer*:ti OR carcinom*:ti OR neoplas*:ti OR tumo*:ti OR adeno*:ti
16. #13 AND #14
17. #15 OR #16
18. 3 and 12 and 17

CNKI and VIP

Search strategy

1. 关键词=糖尿病 or 主题=治疗
2. 关键词=二甲双胍 or 关键词=服用 or 关键词=血糖异常 or 关键词=降糖药
3. 1 or 2
4. 主题=结直肠腺瘤 or 主题=腺瘤性息肉
5. 关键词=结直肠癌 or 关键词=肿瘤
6. 4 or 5
7. 3 and 6

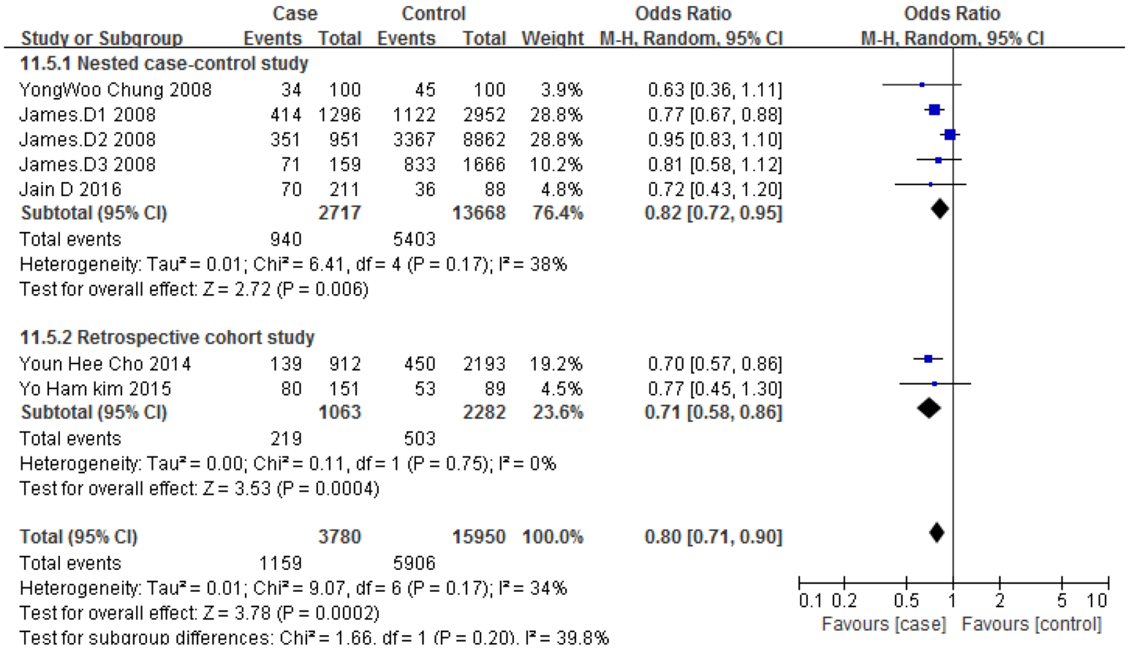

Supplementary Figure 1: Forest plot of the association between metformin therapy and colorectal adenomas – subgroup based on study type.

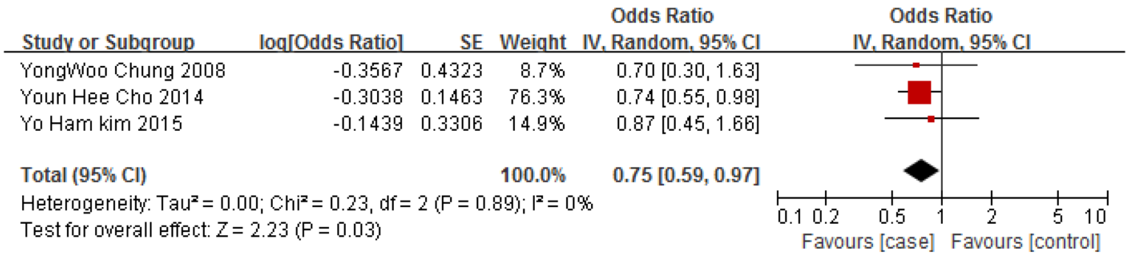

Supplementary Figure 2: Forest plot of the association between metformin therapy and colorectal adenomas – adjusted odds ratio.

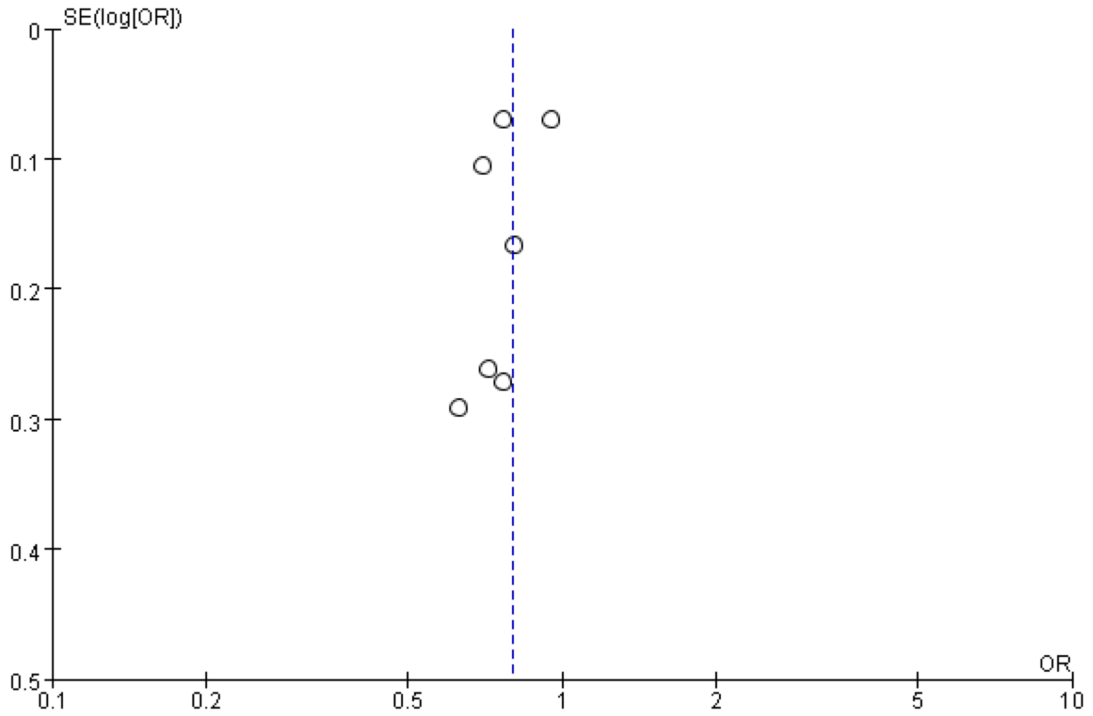

Supplementary Figure 3: Funnel plot of metformin therapy and colorectal adenomas included in meta-analysis.

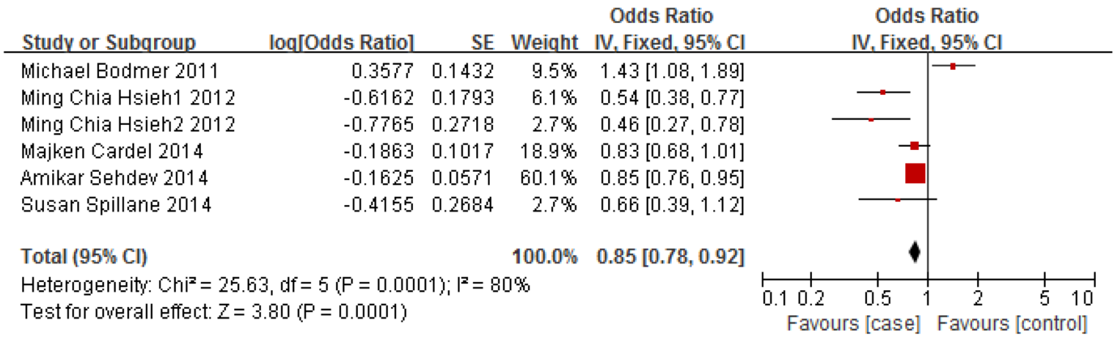

Supplementary Figure 4: Forest plot of the association between metformin therapy and colorectal cancer – adjusted OR.

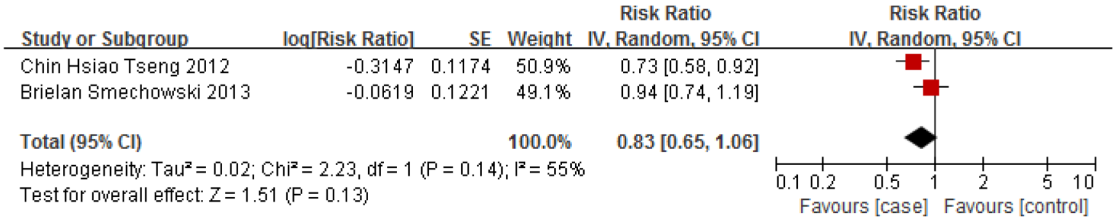

Supplementary Figure 5: Forest plot of the association between metformin therapy and colorectal cancer – adjusted RR.

Study or Subgroup	log[Hazard Ratio]	SE	Weight	Hazard Ratio IV, Random, 95% CI
Gillian Libby 2009	-0.5108	0.233	8.0%	0.60 [0.38, 0.95]
Meei Shyuan 2011	-1.0217	0.5197	2.3%	0.36 [0.13, 1.00]
Rikje Ruiter 2012	-0.0943	0.0171	21.6%	0.91 [0.88, 0.94]
Konstantinos 2014	-0.0834	0.0975	16.7%	0.92 [0.76, 1.11]
Hua Xu1 2015	-0.6931	0.2439	7.5%	0.50 [0.31, 0.81]
Hua Xu2 2015	-0.5108	0.1582	12.1%	0.60 [0.44, 0.82]
Yu ching Chen 2015	-0.6931	0.2606	6.9%	0.50 [0.30, 0.83]
Bernd Kowall1 2015	0.0488	0.1078	15.9%	1.05 [0.85, 1.30]
Bernd Kowall1 2015	-0.0305	0.2122	8.9%	0.97 [0.64, 1.47]
**Total (95% CI)**			**100.0%**	**0.77 [0.66, 0.91]**

Heterogeneity: $Tau^2 = 0.03$; $Chi^2 = 26.21$, df = 8 (P = 0.0010); $I^2 = 69\%$
Test for overall effect: Z = 3.09 (P = 0.002)

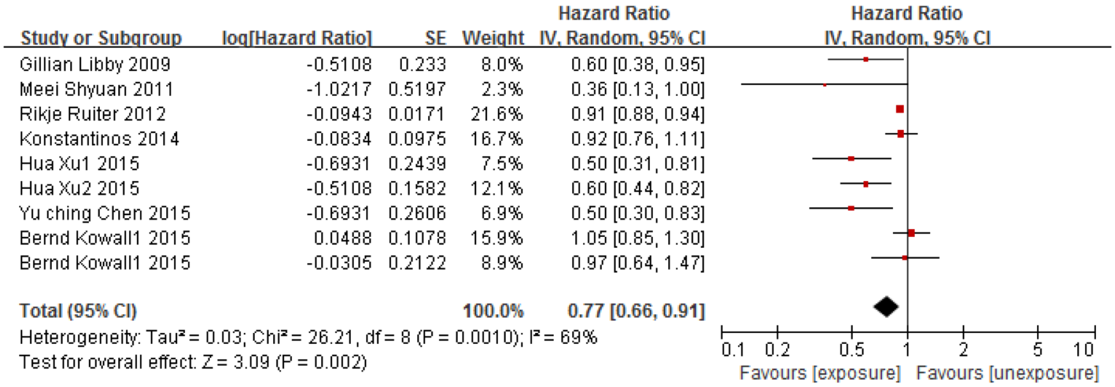

Supplementary Figure 6: Forest plot of the association between metformin therapy and colorectal cancer – adjusted HR.

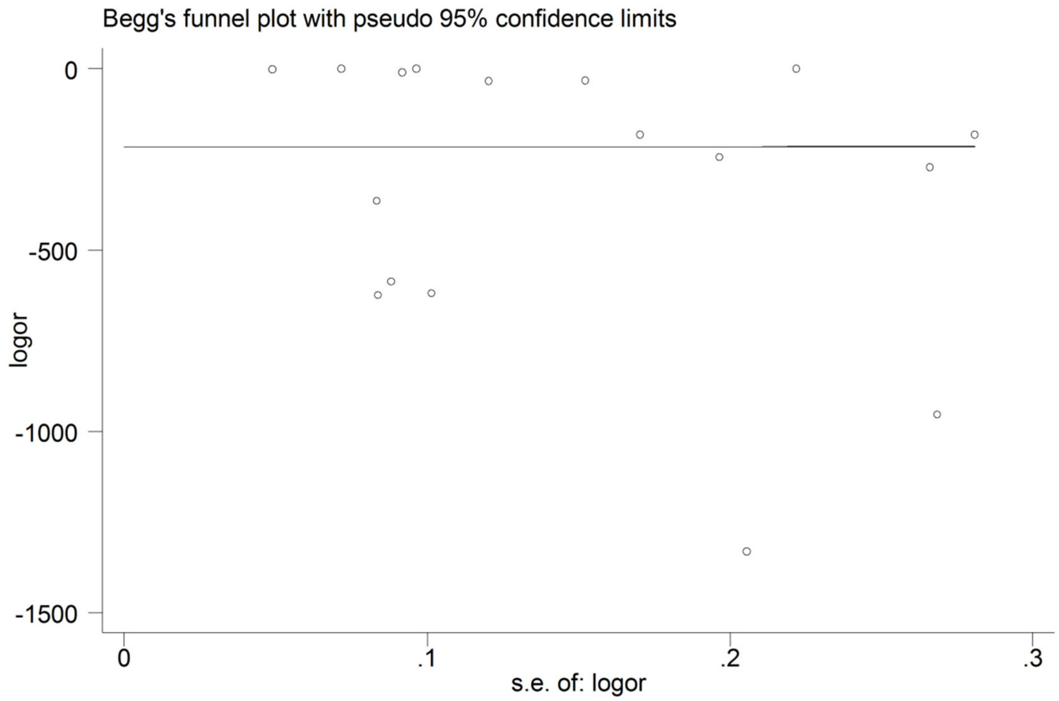

Supplementary Figure 7: Funnel plot of Begg's test.

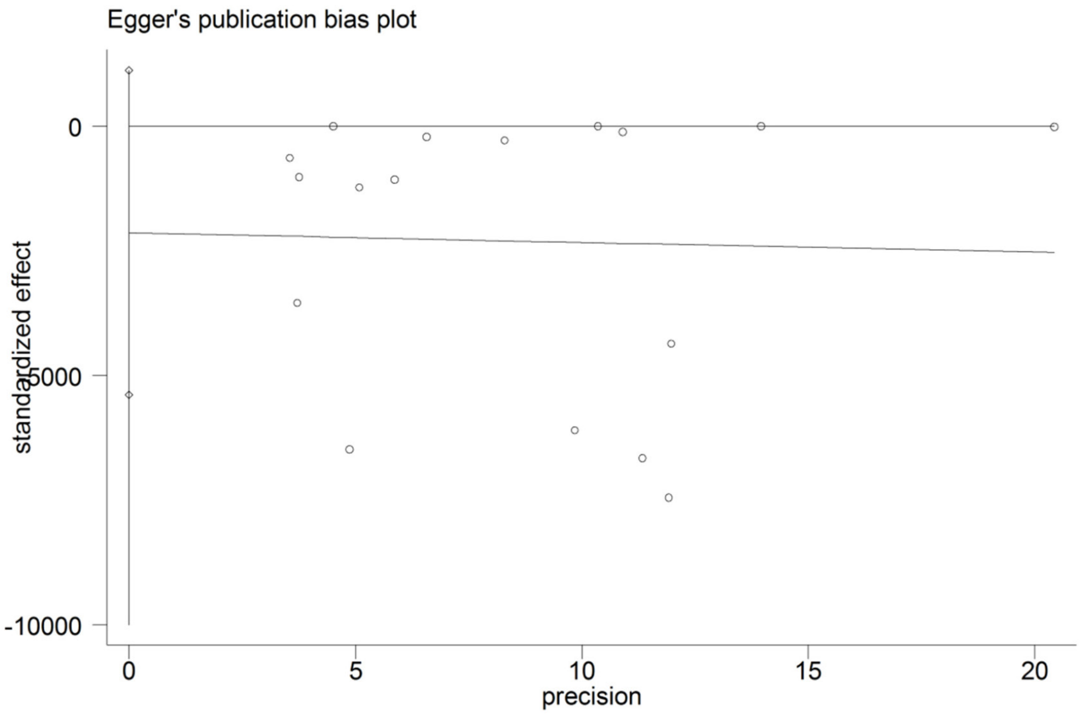

Supplementary Figure 8: Funnel plot of egger’s test.

Supplementary Table 1: Quality assessment of cohort studies (evaluated with NOS)

Author	year	Selection				Comparability	Outcome			Total score
		Item1	Item2	Item3	Item4	Item5	Item6	Item7	Item8	
Gillian Libby [4]	2009	1	1	1	1	2	1	1	1	9
Meei-Shyuan Lee [18]	2011	1	1	1	1	2	1	1	1	9
Rikje Ruiter [5]	2012	1	1	1	1	2	1	1	1	9
Ming-Chia Hsieh [12]	2012	1	1	1	1	2	1	1	1	9
Chin-Hsiao Tseng [11]	2012	1	1	1	1	1	1	1	1	8
Konstantinos [19]	2014	1	1	1	1	2	1	1	1	9
Susan Spillane [20]	2014	1	1	1	1	2	1	1	1	9
Youn Hee Cho [10]	2014	1	1	1	1	2	1	1	1	9
Yo Ham Kim [9]	2015	1	1	1	1	2	1	1	1	9
Hua Xu [21]	2015	1	1	1	1	2	1	1	1	9
Yu Ching Chen [13]	2015	1	1	1	1	2	1	1	0	8
Bernd Kowall [22]	2015	1	1	1	1	2	1	1	1	9

Item1: Representativeness of the exposed cohort
Item2: Selection of the non-exposed cohort
Item3: Ascertainment of exposure
Item4: Demonstration that outcome of interest was not present at start of study
Item5: Comparability of cohorts on the basis of the design or analysis
Item6: Assessment of outcome
Item7: Was follow-up long enough for outcomes to occur
Item8: Adequacy of follow up of cohorts

Supplementary Table 2: Quality assessment of case-control studies (evaluated with NOS)

Author	year	Selection				Comparability	Exposure			Total score
		Item1	Item2	Item3	Item4	Item5	Item6	Item7	Item8	
Yong Woo Chung [15]	2008	1	1	1	1	1	1	1	1	8
James D [23]	2008	1	1	1	1	1	1	1	1	8
Michael Bodmer [24]	2011	0	1	1	1	2	1	1	1	8
Brielan Smechowski [25]	2013	0	1	1	1	2	1	1	1	8
Majken Cardel [14]	2014	1	1	1	1	1	1	1	0	7
Amikar Sehdev [26]	2014	1	1	1	1	2	1	1	0	8
Jain D [27]	2016	1	1	1	1	1	1	1	0	7

Item1: Is the case definition adequate?
Item2: Representativeness of the cases
Item3: Selection of Controls
Item4: Definition of Controls
Item5: Comparability of cases and controls on the basis of the design or analysis
Item6: Ascertainment of exposure
Item7: Same method of ascertainment for cases and controls
Item8: Non-Response rate
